# Internal post-bariatric hernia due to hepatic adhesion: a case report

**DOI:** 10.31744/einstein_journal/2023RC0478

**Published:** 2023-08-28

**Authors:** Bruno Mirandola Bulisani, Murilo Rocha Rodrigues, Luiz Guilherme Lisboa Gomes, Milena Arruda de Oliveira Leite, Felipe Martin Bianco Rossi, Nathan Rostey, Jaques Waisberg

**Affiliations:** 1 RR Médicos Cirurgiões São Bernardo do Campo SP Brazil RR Médicos Cirurgiões, São Bernardo do Campo, SP, Brazil.; 2 Centro Universitário FMABC Santo André SP Brazil Centro Universitário FMABC, Santo André, SP, Brazil.

**Keywords:** Bariatric surgery, Gastric bypass, Anastomosis, Roux-en-Y, Hernia, obturator, Incisional hernia, Postoperative complications

## Abstract

Roux-en-Y gastric bypass, a procedure proven effective for treating morbid obesity and metabolic disorders, carries the risk of complications such as the formation of internal hernias. These hernias are often difficult to diagnose and can be potentially fatal because they can cause structural obstruction. Most internal hernias occur in the jejunojejunostomy mesentery space, followed by Petersen's space hernias, although herniation at other locations can also occur. Our case report presents an example of a rare internal hernia after laparoscopic Roux-en-Y gastric bypass. A 36-year-old woman presented with an uncommon internal hernia located between the liver and alimentary loop, resulting in the formation of a new space and consequently incarcerating the entire biliopancreatic loop. This type of internal hernia is rare and has not been reported in the literature, indicating that this is the first report of such a case. In this case, we realized that the diagnosis was challenging and imaging examinations could not help determine the etiology of the pain and obstruction. Therefore, videolaparoscopy revealed an uncommon hernia formed by firm adhesion between the hepatic segment III and the alimentary loop mesentery. Our case is an example of an internal hernia that was not detected with a normal computed tomography scan of the abdomen and pelvis. Only diagnostic laparoscopy revealed herniation, effectively preventing further complications for the patient.

## INTRODUCTION

Roux-en-Y gastric bypass, which typically involves a small gastric pouch, a 50cm biliary limb, and a 150cm alimentary limb, has emerged as one of the most effective solutions for morbid obesity and metabolic disorders.^(1,2)^ The major differences between open and laparoscopic Roux-en-Y gastric bypass are the exposure and method of access. Early complications associated with this surgery include anastomotic leak, pulmonary embolism, wound infection, gastrointestinal hemorrhage, and mortality due to respiratory insufficiency. Late complications may include incisional hernia, bowel obstruction, internal hernia, stomal stenosis, and marginal ulcers.^(3,4)^

A study by Chang et al. revealed that hernias develop in 31% of all cases of postoperative intestinal obstructions following Roux-en-Y gastric bypass.^(5)^ Internal herniation occurs in approximately 6% of patients and can be attributed to significant weight loss or the inability to close internal spaces, with major symptoms including abdominal pain, nausea, and vomiting. Computed tomography (CT) is the optimal method for investigating hernias, and the primary risk factors are small bowel ischemia and necrosis; therefore, surgical investigation should be considered in these cases.^(3,5)^ In this case report, we present an uncommon internal hernia that formed between the liver and the alimentary loop, creating a new space and subsequently resulting in the incarceration of the biliopancreatic loop.

## CASE REPORT

**Figure 1:**
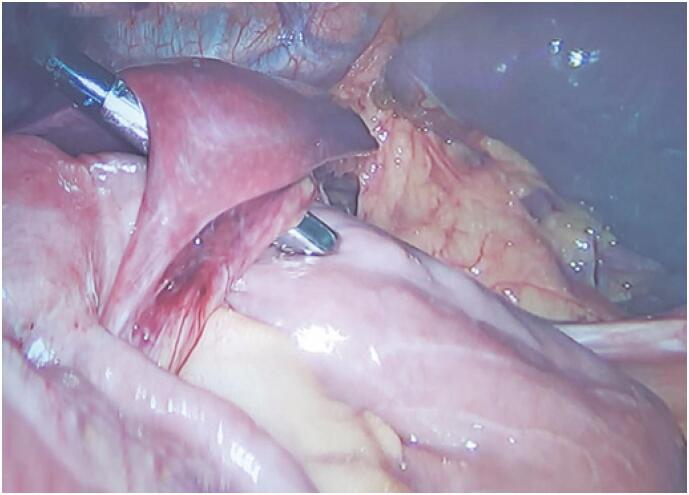
Unusual hernia formed by a firm adhesion between hepatic segment III and the alimentary loop mesentery

Two years ago, a 36-year-old woman with morbid obesity and metabolic syndrome underwent vertical sleeve gastrectomy at our center, located in Santo André, Brazil. Following this procedure, the patient underwent revision laparoscopic Roux-en-Y gastric bypass for reflux disease. Four months after the second surgery, she presented to the emergency department with acute mesogastric abdominal postprandial pain, which had persisted for three consecutive days, accompanied by nausea and emesis. Physical examination revealed a soft abdomen without sudden decompression but with diffuse tenderness in the mesogastric region. Laboratory results were normal. Both CT and upper digestive endoscopy were performed but showed normal results. Consequently, videolaparoscopy was employed as a diagnostic method. Imaging revealed an uncommon internal hernia formed by a new space resulting from firm adhesion between hepatic segment III and the alimentary loop mesentery. The biliopancreatic loop was almost completely incarcerated, constituting an internal hernia and significant distension upstream of the alimentary loop without intestinal distress (Figure 1). Laparoscopic lysis of this adhesion was performed, resulting in immediate resolution of the obstructive condition and improvement in distension. The patient showed clinical improvement, reported no pain, had good dietary acceptance, and was discharged on the first postoperative day.

This case study was approved by the Ethics Committee of *Centro Universitário FMABC* (CAAE: 63760922.1.0000.0082, # 5.845.049).

## DISCUSSION

Sleeve gastrectomy and gastric bypass are currently among the most commonly performed gastrointestinal operations, with a very low 90-day mortality rate of less than 0.5%.^(6,7)^ Possible complications include the formation of an internal hernia, which can be challenging to diagnose and potentially fatal because it can cause obstruction.^(3)^ More than half of the internal hernias occur in the jejunojejunostomy mesentery, followed by Petersen's space hernia. When it presents with herniated limb, it can be twisted accompanied by the rotation of the mesenteric, resulting in venous and lymphatic engorgement and sometimes causing bowel obstruction.^(8,9)^

In this case report, the patient presented with an uncommon internal hernia located between the liver and alimentary loop, forming a new space and consequently incarcerating the whole biliopancreatic loop. This type of internal hernia is rare and has not been reported in the literature, indicating that this is the first reported case of its kind. In this case, we realized that the diagnosis was challenging and imaging examinations could not help determine the etiology of the pain and obstruction. Videolaparoscopy was indicated, which helped diagnose and treat the patient. We introduced a 10-12mm optical trocar in the standard manner via an umbilical incision, and two 5mm laparoscopic ports were positioned in the right flank (right lower quadrant and subcostal positions) and one 5mm laparoscopic port on the left flank (subcostal position). It revealed the presence of an internal post-bariatric hernia caused by firm hepatic adhesion to the alimentary limb that was not evident on the CT scan performed before surgery. An unusual internal hernia was formed by the firm adhesion between hepatic segment III and the alimentary loop mesentery, creating a new space in which the entire bilar limb was incarcerated but with no signs of intestinal ischemia. Lysis of the adhesion was performed, and the bilateral limb returned to its normal position and normal peristalsis to resolve the obstruction. The patient tolerated the procedure well and remained stable upon discharge on the first postoperative day.

Our case is an example of the diagnosis of an internal hernia that involved abdominal pain after laparoscopic Roux-en-Y gastric bypass surgery. Despite normal abdominal and pelvic computed tomography scans, only diagnostic laparoscopy revealed herniation. Therefore, it is necessary to consider the diagnosis and persistence of clinical symptoms, even in the presence of normal examination results, warranting the consideration of diagnostic videolaparoscopy due to the risk of this pathology. Although this type of internal hernia is rare and has not been previously reported, it should be included in our diagnostic hypotheses due to the possibility of adhesion formation related to the proximity of the left hepatic lobe to the gastroenteroanastomosis and alimentary loop.

## CONCLUSION

Our case is an example of the diagnosis of an internal hernia that involved abdominal pain after laparoscopic Roux-en-Y gastric bypass surgery. Despite normal abdominal and pelvic computed tomography scans, only diagnostic laparoscopy revealed herniation. Due to the non-specific symptoms and potentially late presentation, diagnosing internal hernias can be challenging and pose risks to the patient, including potential ischemia and necrosis of the structures. Lytic surgery can serve as the primary treatment for these patients.
